# Awareness About Folic Acid Supplementation in First-Trimester Pregnant Women of Rural Raipur District, Chhattisgarh, and Its Determinants: A Cross-Sectional Study

**DOI:** 10.7759/cureus.40583

**Published:** 2023-06-18

**Authors:** Anjali Pal, Arvind K Shukla, Archismita Santra, Abhiruchi Galhotra, Pushpawati Thakur, Suprava Patel, Sunita Singh, Sarita Rajbhar

**Affiliations:** 1 Community and Family Medicine, All India Institute of Medical Sciences, Raipur, Raipur, IND; 2 Community and Family Medicine/Biostatistics, All India Institute of Medical Sciences, Raipur, Raipur, IND; 3 Obstetrics and Gynaecology, All India Institute of Medical Sciences, Raipur, Raipur, IND; 4 Biochemistry, All India Institute of Medical Sciences, Raipur, Raipur, IND; 5 Pediatric Surgery, All India Institute of Medical Sciences, Raebareli, Raebareli, IND

**Keywords:** maternal serum screening tests, neural tube defects, vitamin b12 deficiency, pregnancy, folate deficiency, folic acid supplementation

## Abstract

Background: Women are supplemented with folic acid (FA) during pregnancy as well as preconceptionally to prevent neural tube defects (NTDs) in newborns. To understand the importance of FA supplementation, women need to have awareness about the same, which in turn may be influenced by different factors. It is also known that both FA and vitamin B12 deficiency tend to cause NTDs in newborns and anemia. Very few studies have studied the relationship between hemoglobin, FA, and vitamin B12 levels. In this study, we aim to estimate the level of awareness of FA supplementation among pregnant women in the first trimester of pregnancy and the factors determining the presence of awareness regarding the same. Also, we aim to estimate any correlation between hemoglobin, FA, and vitamin B12 levels among a subset of pregnant women.

Methods: A cross-sectional study was conducted in the Abhanpur Block of Raipur district in Chhattisgarh among 399 pregnant women in their first trimester of pregnancy, in which their knowledge was assessed using a pretested semistructured questionnaire. Each participant's knowledge score regarding FA supplementation was calculated and scored based on six indicators and classified as low, intermediate, and high scores. Logistic regression was applied to find out any significant association between knowledge about FA supplementation with any other sociodemographic variables. Scatter plots were used to assess the correlation of FA with hemoglobin, vitamin B12, and knowledge scores among 104 participants.

Results: The majority (77.9%) of women had low knowledge scores with a mean score of 1.4 (0.15). It was found that only 45.6% of the participants knew the importance of FA supplementation, and the majority (23.1%) were informed by auxiliary nurse midwives (ANMs) followed by doctors. The majority (41.6%) of the study participants also did not know when to start FA, and only 1.3% knew that FA should be taken preconceptionally. On multivariable logistic regression, women who lived in joint families had significantly higher odds of having intermediate knowledge compared to those who lived in nuclear families. Although not statistically significant, there was a positive correlation between serum vitamin B12 and FA levels and also between hemoglobin and serum FA levels. However, a significant positive correlation was found between serum FA levels and the knowledge scores of the study participants.

Conclusion: The majority of study participants had poor knowledge and awareness regarding FA supplementation. So, health education, as well as information, education, and communication (IEC) activities, is required to improve the knowledge about FA supplementation among women of reproductive age in the community. A better understanding of FA supplementation can lead to adherence to FA consumption and prevent NTDs among newborns.

## Introduction

Neural tube defects (NTDs) are an important preventable cause of morbidity and mortality worldwide [1]. The majority of pregnant females and healthcare providers tend to ignore folic acid (FA) supplementation in the first trimester [2]. Very few studies have been conducted in India on the awareness of pregnant women regarding FA supplementation, providing comprehensive information about their current knowledge and its determinants [3,4]. Globally, the prevalence of NTDs among newborns is approximately 1-5/1000 live births with a 2%-3% risk of recurrence [5]. According to a systematic review by Bhide et al., the overall prevalence of NTDs at birth in India has been reported to be 4.1/1000 (95% confidence interval [CI], 3.1-5.4), and the live birth and stillbirth prevalence of NTDs was found to be 1.3/1000 births (95% CI, 0.9-1.8) and 1.7/1000 births (95% CI, 0.7-4.0), respectively [6]. NTDs are preventable if FA is supplemented preconceptionally as well as during pregnancy, and more than 50% of NTDs may be prevented by this intervention [7].

According to the Reproductive, Maternal, Neonatal, Childhood Health Plus Adolescent (RMNCH+A) strategy, women having planned pregnancies should start taking 400 μg of FA daily three months before conception [8]. Intensified Iron Plus Initiative (I-NIPI) also recommends iron and folic acid (IFA) supplementation during pregnancy, with 60 mg of elemental iron and 500 μg FA [9]. There has been less focus on vitamin B12 deficiency, although it is also a known cause of anemia and about 40%-70% of women in India suffer from vitamin B12 deficiency during pregnancy [10]. Recently, studies suggested that deficient or inadequate vitamin B12 or an imbalance between serum folate and vitamin B12 levels can also lead to NTDs [10,11]. Accredited social health activists (ASHAs), auxiliary nurse midwives (ANMs), and medical officers are supposed to spread awareness in the community regarding FA supplementation and its importance [8]. Hence, the timely dispersion of knowledge about FA supplementation in the community by health providers is crucial in reducing the burden of NTDs among newborns and determining the success of the RMNCH+A program. There has been no such study in Chhattisgarh and eastern India, which assessed the awareness regarding FA supplementation among pregnant women. Furthermore, very few studies have estimated serum FA and vitamin B12 levels among pregnant women and assessed the correlation between them [4,11].

In this study, we aim to estimate the level of awareness of folic acid supplementation among pregnant women in their first trimester of pregnancy and the factors determining their awareness of FA supplementation. Also, as a secondary objective, the levels of hemoglobin, serum FA, and vitamin B12 among a subset of these pregnant women were measured, and the correlation of serum FA levels with hemoglobin, serum vitamin B12, and knowledge scores of the participants were assessed.

## Materials and methods

A cross-sectional study was conducted from March to October 2019 in the Abhanpur Block of Raipur district after obtaining ethical approval from the Institute Ethics Committee of All India Institute of Medical Sciences (AIIMS), Raipur. The Abhanpur Block has two community health centers (CHCs), seven primary health centers (PHCs), and 37 subcenters (SCs). Based on the presumed prevalence of knowledge of FA supplementation as 50%, an absolute precision of 5% at a 95% confidence interval, and a nonresponse rate of 10%, a sample of 429 pregnant women was estimated to be required because no previous data on the mentioned indicator was available previously from the region.

Pregnant women in their first trimester were eligible for participating in this study. They were recruited from the regularly organized antenatal clinics at the PHCs of the block. The selection of study participants from each PHC was done by probability proportional to size sampling after the complete enumeration of the pregnant women in the first trimester of pregnancy under each PHC. More samples were selected from PHCs that had a greater number of pregnant women in the first trimester of pregnancy, and women were selected randomly from each PHC in subsequent antenatal clinics. A total of 399 women were recruited for this study.

Written informed consent was obtained from all the study participants before the interview. Data were collected using a predesigned and pretested semistructured questionnaire validated by three experts, which was administered by the study investigators and field staff after one day of questionnaire orientation.

The questionnaire for the pregnant women included information regarding their sociodemographic details, history of previous pregnancies, number of living children, awareness of FA supplementation and its importance, the current practice of FA intake, and the history of starting preconceptional FA supplementation.

Any women who were found to be seriously ill and unable to comprehend the instructions of the interviewer or who have diarrhea, steatorrhea, or hyperemesis were excluded as these diseases cause increased excretion of FA from the body and therefore cause variation in the serum folic acid level. Serum FA, serum vitamin B12, and hemoglobin levels were assessed among 104 study participants who were selected through systematic random sampling after their voluntary informed consent. A blood sample of 5 ml was collected under stringent aseptic conditions in a sterile and plain vacutainer and carried to AIIMS Raipur while maintaining a proper cold chain. The sample was centrifuged, and the serum was separated and stored at -20°C till the biochemical tests were applied. Serum folate and vitamin B12 were assessed by the chemiluminescence method, and hemoglobin was assessed by an auto-analyzer in the department of biochemistry of AIIMS, Raipur. The results of all the tests were communicated to the respective pregnant women, and they were referred to the obstetrics and gynecology department at AIIMS Raipur for further follow-up antenatal care.

Statistical analysis was done using Stata version 12.0 (StataCorp LP, College Station, TX). The categorical variables were presented in the form of frequency and percentages. A knowledge score was computed based on six indicators: (1) if participants ever heard about FA, (2) if they knew that FA is required during pregnancy, (3) if they knew that FA prevents NTDs among newborns, (4) if they knew that FA supplementation prevents other congenital defects among newborns, (5) if they knew that it prevents anemia, and (6) if they knew that it should be taken periconceptionally.

These indicators were adopted from a similar study conducted by Saxena et al. and scored accordingly [3]. Each correct response was awarded a score of one, and each wrong response was given a score of zero; thus, the maximum possible score of the indicators for each participant was six. The final score for each participant was obtained by summating the scores for every indicator. Based on this, women scoring ≤2 were considered to have low knowledge, while women scoring more than 4 were considered to have high knowledge. The rest of the women scoring 3-4 were considered to have intermediate knowledge.

Categorical variables were presented in the form of frequencies and percentages. Quantitative variables were presented in the form of mean and standard error. Logistic regression was done to assess the factors associated with the knowledge of the participants. To adjust for confounders, multivariable logistic regression was done on the factors for which the p-value was ≤0.2 in univariable logistic regression. A p-value less than 0.05 in the multivariable logistic regression model was considered to be significant. Pearson’s correlation was performed, and scatter plots were presented between FA levels and hemoglobin, vitamin B12, and knowledge scores of 104 study participants to assess any correlation, and linear regression was done to find the association between them.

## Results

The mean age of the participants was 23.4 years (0.15). The mean knowledge score of the study participants was 1.4 (SE 0.05, 95% CI: 1.2-1.5). It was found that the majority of participants (77.9%) had low knowledge scores on FA supplementation, 19.3% had intermediate scores, and none had high knowledge scores (Table 1).

**Table 1:** Distribution of study participants according to knowledge scores (n = 399)

Knowledge score	Number of pregnant women (%)
Low score (0-2)	311 (77.9)
Intermediate score (3-4)	88 (22.1)
High score (>4)	0 (0.0)

Most of the study participants (58.1%) belonged to the age group of 21-25 years and were educated above high school (70.7%) though only 46.6% were employed. However, the husbands of most of the participants (42.3%) were educated up to eighth grade. About 46.9% of the study participants were primigravida, and 91.7% had planned their pregnancy. It was also found that only 199 out of 399 (49.8%) women were taking FA during pregnancy. Out of 199 women who took FA during pregnancy, 72 (50.7%) of them had a previous history of childbirth, and 179 (89.9%) of them had planned their pregnancy (Table 2).

**Table 2:** Distribution of study participants’ knowledge score and practices on folic acid supplementation according to sociodemographic variables (n = 399) FA: Folic acid.

Sociodemographic variables		Total (n = 399)		Knowledge score ≥ 3 (n = 88)		FA taken during pregnancy (n = 199)	
		N	%	N	%	N	%
Age (years)	≤20	76	19.0	12	13.6	30	15.1
	21-25	232	58.2	53	60.2	121	60.8
	26-30	81	20.3	22	25.0	44	22.1
	>30	10	2.5	1	1.2	4	2.0
Education of the participant	Illiterate	19	4.8	2	2.3	12	6.0
	Up to 8^th^ grade	98	24.6	21	23.9	43	21.6
	High school	126	31.6	27	30.7	60	30.2
	Intermediate	117	29.3	26	6.5	59	29.6
	Graduate and above	39	9.7	12	13.6	25	12.6
Husband’s education	Illiterate	16	4.0	2	18.2	8	4.0
	Up to 8^th^ grade	169	42.4	44	50.0	83	41.7
	High school	97	24.4	18	20.5	42	21.1
	Intermediate	61	15.2	14	15.9	32	16.1
	Graduate and above	56	14.0	10	11.4	34	17.1
Occupation	Employed	186	46.7	41	46.6	92	46.2
	Unemployed	213	53.3	47	53.4	107	53.8
Religion	Hindu	396	99.2	87	98.9	197	99.0
	Muslim	3	0.8	1	1.1	2	1.0
Family type	Nuclear	66	16.5	10	11.4	29	14.6
	Joint	333	83.5	78	88.6	170	85.4
Previous delivery	No (Primigravida)	187	46.8	35	39.8	98	49.2
	One	154	35.7	38	43.2	72	36.2
	More than one	58	14.5	15	17.0	29	14.6
Number of children alive	No	198	49.6	37	42.0	101	50.7
	One	150	37.6	37	42.0	71	35.8
	More than one	51	12.8	14	16.0	27	13.5
Planned pregnancy	Yes	366	91.7	80	90.9	179	90.0
	No	33	8.3	8	9.1	20	10.0

About 38.8% of the pregnant women reported that they were not advised to take FA by anyone. Only 22% of the participants stated that they were advised to take FA by doctors. Though 244 out of 399 women (61.1%) had heard about FA supplementation, 182 women (54.4%) reported that they knew the importance of taking FA during pregnancy. However, 98 of the 182 participants (53.8%) who stated to have known the importance of taking FA did not remember it. Only 1.3% of the women knew that FA should be taken before pregnancy, while 41.6% did not know when to start FA supplementation (Table 3).

**Table 3:** Distribution of pregnant women according to the indicators of knowledge assessment on FA supplementation (n = 399) FA: Folic acid; ANM: Auxiliary nurse midwife; ASHA: Accredited social health activist.

Indicators		Number	%
Heard about FA	Yes	244	61.1
	No	155	38.9
Who advised taking FA during pregnancy?	Doctor	90	22.6
	ANM	92	23.1
	ASHA	49	12.3
	Relative/friends	10	2.5
	Others	3	0.8
	None	155	38.8
Knew the importance of FA supplementation during pregnancy	Yes	182	45.6
	No	217	54.4
If yes, the importance of taking FA (n = 182)	Helps ensure women have a healthy baby	84	46.1
	Can’t remember	98	53.8
Knew when FA supplementation should be started	FA should be started prior to conception	5	1.3
	FA should be started after the first trimester of pregnancy	113	28.3
	FA should be taken during early pregnancy	115	28.8
	Do not have any idea/don’t know	166	41.6

On multivariable logistic regression, women who lived in a joint family had significantly higher odds of having intermediate knowledge about FA supplementation compared to those who lived in nuclear families (AOR: 2.2, 95% CI: 1.1-4.6, p-value: 0.04) (Table 4).

**Table 4:** Bivariable and multivariable logistic regression to assess the factors associated with low and intermediate knowledge scores of study participants regarding FA supplementation COR: Crude odds ratio; AOR: Adjusted odds ratio; 95% CI: 95% confidence interval. Factors adjusted in multivariable logistic regression are husband's education, type of family, history of previous deliveries, and the number of living children. (These had p-value ≤ 0.2 in bivariable logistic regression.)

Independent variables	Low knowledge score, N (%)	Intermediate knowledge score, N (%)	COR, (95% CI)	p-value	AOR, (95% CI)	p-values
Age category (in years)						
≤25 years	243 (78.1)	65 (73.9)	Reference			
>25 years	68 (21.9)	23 (26.1)	1.3 (0.7-2.2)	0.4		
Education						
Below high school	94 (30.2)	23 (26.1)	Reference			
High school and above	217 (69.8)	65 (73.9)	1.2 (0.7-2.0)	0.4		
Husband’s education						
Below high school	139 (44.7)	46 (52.3)	Reference			
High school and above	172 (55.3)	42 (47.7)	0.7 (0.5-1.1)	0.2	0.7 (0.4-1.1)	0.2
Occupation						
Employed	145 (46.6)	41 (46.6)	Reference			
Unemployed	166 (53.4)	47 (53.4)	1.0 (0.6-1.6)	0.9		
Religion						
Hindu	309 (99.4)	87 (98.9)	Reference			
Muslim	2 (0.6)	1 (1.1)	1.7 (0.2-19.8)	0.6		
Family type						
Nuclear	56 (18.0)	10 (11.4)	Reference			
Joint	255 (82.0)	78 (88.6)	1.7 (0.8-3.5)	0.1	2.2 (1.1-4.6)	0.04
Previous deliveries						
None	152 (48.9)	35 (39.8)	Reference			
One or more	159 (51.1)	33 (60.2)	1.4 (0.8-2.3)	0.1	1.0 (0.2-5.0)	0.9
Living children						
None	161 (51.8)	37 (42.0)	Reference			
One or more	150 (48.2)	51 (58.0)	1.5 (0.9-2.3)	0.1	1.5 (0.3-7.5)	0.6
Planned pregnancy						
Yes	286 (92.0)	80 (90.9)	Reference			
No	25 (8.0)	8 (9.1)	1.1 (0.5-2.6)	0.7		

**Figure 1:**
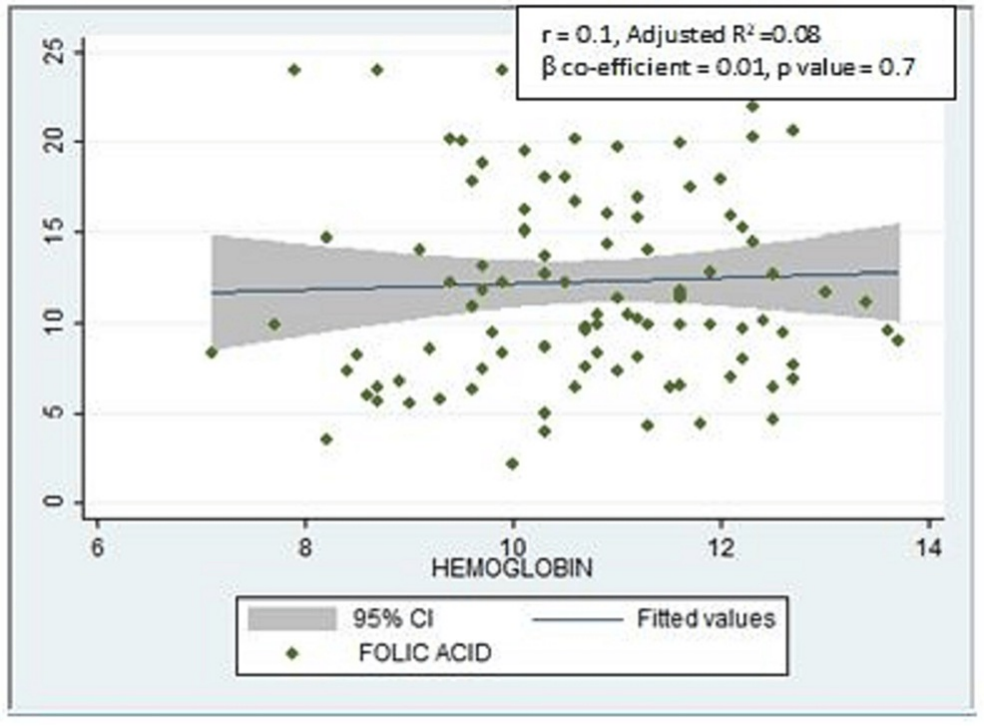
Scatter plot showing the correlation between serum folic acid and hemoglobin levels (n = 104)

There was a positive correlation between serum FA levels and hemoglobin, which was not statistically significant (r = 0.1, adjusted R2 = 0.08, ꞵ coefficient = 0.01, p-value = 0.7) (Figure 1).

**Figure 2:**
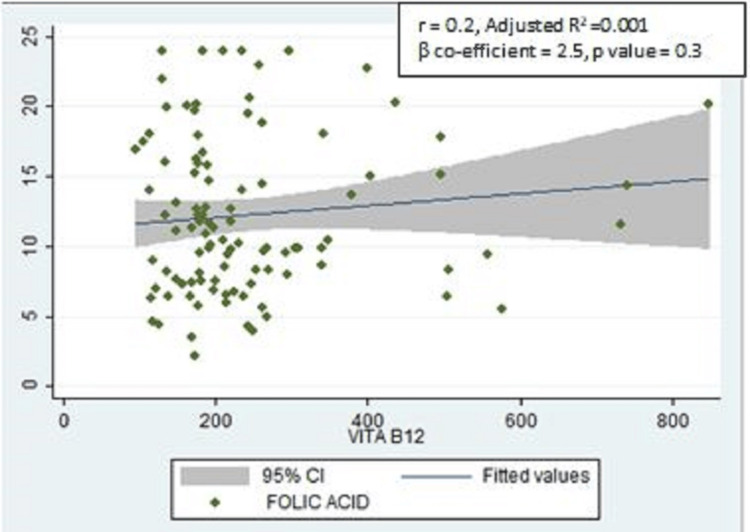
Scatter plot showing the correlation between serum folic acid and serum vitamin B12 levels (n = 104) VITAB12: Serum vitamin B12.

There was also a positive correlation between the serum FA levels and serum vitamin B12 levels, but it was not statistically significant (r = 0.2, adjusted R2 =0.001, ꞵ coefficient = 2.5, p-value = 0.3) (Figure 2). 

**Figure 3:**
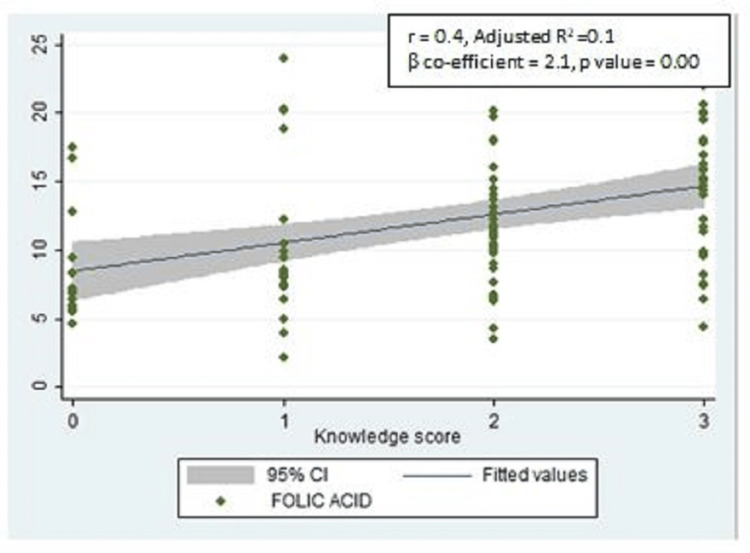
Scatter plot showing the correlation between serum folic acid and knowledge scores of the study participants regarding FA supplementation (n = 104) FA: Folic acid.

However, there was a significant positive correlation between the serum FA levels and the knowledge scores of the study participants (r = 0.4, adjusted R2 = 0.1, ꞵ coefficient = 2.1, p-value = 0.00) (Figure 3).

## Discussion

In this study, it was found that the majority of the study participants (77.9%) had low knowledge regarding FA supplementation before and during pregnancy, even if the majority of them were educated above high school. Though the majority of the pregnant women had heard about FA and stated that they knew the importance of FA supplementation, which might be due to social desirability bias, most of them could not remember the importance of taking FA. Very few women (1.3%) knew that FA supplementation is required before pregnancy even when a majority of them had planned pregnancy, while only 28.8% of the women knew FA should be taken during early pregnancy. Similar findings have been noticed in several studies globally as well as in India [3,12-16].

It has also been observed in the study that most of the pregnant women were not advised to take FA tablets during pregnancy by healthcare workers, and only 22.6% of doctors had advised them to do so. According to the RMNCH+A strategy, ASHAs are responsible for spreading awareness in the community regarding FA supplementation. However, only 12.3% of the women reported that they were advised to take FA during pregnancy and preconceptionally by ASHAs. Thus, there remains a gap in communication between healthcare workers with beneficiaries, which leads to overall low knowledge scores. It was found in the current study that women who lived in joint families had significantly higher knowledge scores on FA supplementation compared to those living in nuclear families. This can possibly be attributed to the sharing of knowledge and experiences among family members who live in a joint family, which resulted in better knowledge scores.

In a study conducted in Northern India among pregnant women by Saxena et al., there was a significant association of knowledge and practices regarding FA supplementation with the level of education of the participants but not with any other sociodemographic variables [3]. In our study, no association of knowledge was found with the education of the participants. This might be because of the difference in the level of education of the participants in both studies. The knowledge of the pregnant women could have been also influenced by their husbands, the majority of whom were educated up to the eighth grade.

Although not statistically significant, a positive correlation was found in our study between serum FA levels and hemoglobin. A similar positive correlation between the serum FA and hemoglobin was also found in a study conducted among women of childbearing age in Senegal, which was statistically significant [17]. This difference in statistical significance may be because of the smaller sample size of our study compared to the above-mentioned study as the measurement of serum folate, hemoglobin, and vitamin B12 was not our primary objective. A positive correlation was found in our study between the serum FA levels and vitamin B12 levels, but it was also not statistically significant, which can again be attributed to a smaller sample size for biochemical tests. A significant positive correlation was also found between the serum FA levels and the knowledge scores among 104 participants for whom serum FA levels were assessed. This reflects the importance of knowledge dissemination on FA supplementation as women who are aware of the importance of FA adhere to FA consumption.

The strength of this study is that we assessed the knowledge on FA supplementation as well as correlated FA levels with other biochemical predictors like hemoglobin and vitamin B12. However, the limitations of the study include a smaller sample size for biochemical tests due to logistic issues. Also, the estimation of knowledge of healthcare providers including ASHAs and ANMs could have helped to address the gaps in spreading awareness of FA supplementation in the community.

## Conclusions

Therefore, we can conclude from this study that though the majority of pregnant women were educated and had planned pregnancies, they were not aware of the importance of taking FA supplementation. It was also found that there have been gaps in advising FA to pregnant women by healthcare workers. Also, serum FA levels were positively correlated with knowledge scores on FA supplementation.

As a result, we recommend that future studies be conducted to assess the knowledge of healthcare workers on FA supplementation and to make efforts to improve their knowledge. Furthermore, health education and discussions may be regularly arranged at antenatal clinics on the importance of FA supplementation and the prevention of NTDs as well as in the community on Village Health and Nutrition Days (VHNDs) among women of reproductive age. This will help to increase the knowledge and practices of women on FA supplementation and ultimately help in the reduction of the burden of NTDs among newborns.
